# Benefits of using intrathecal buprenorphine

**Published:** 2014

**Authors:** Seyed Mozaffar Rabiee, Ebrahim Alijanpour, Ali Jabbari, Sara Rostami

**Affiliations:** 1 Department of Anesthesiology and Intensive Care, Babol University of Medical Sciences, Babol, Iran; 2 Department of Anesthesiology and Intensive Care, Golestan University of Medical Sciences, Gorgan, Iran; 3 Babol University of Medical Sciences, Babol, Iran

**Keywords:** Buprenorphine, Intrathecal, Spinal Anesthesia, Post operation, Pain

## Abstract

***Background: ***General anesthesia draws attention to the most commonly used modalities for post cesarean delivery pain relief in systemic administration of opioids, while the administration of small dose of intrathecal opioid during spinal anesthesia can be a possible alternative. The aim of this study was to evaluate the effects of buprenorphine on cesarean section prescribed intrathecally.

***Methods:*** This double blind randomized clinical trial study was conducted in patients for cesarean section under spinal anesthesia. The patients were randomly divided into case and control groups. Case group (208 patients) received 65-70 mg of 5% lidocaine plus 0.2 ml of buprenorphine while the same amount of 5% lidocaine diluted with 0.2 ml of normal saline was given to 234 cases in the control group. Hemodynamic changes and neonatal APGAR scores (Appearance, Pulse, Grimace, Activity, Respiration) were recorded. Pain score was recorded according to the visual analog scale. This study was registered in the Iranian Registry of clinical Trials; IRCT2013022112552N1.

***Results:*** The mean age of case and control groups was 24.4±5.38 and 26.84±5.42 years, respectively. Systolic blood pressure was not significantly different until the 45th minute but diastolic blood pressure showed a significant difference at the 15th and the 60th minutes (P<0.001). Heart rate changes were significantly different between cases and controls at the initial 5th, 15th and after 60th minutes (P<0.001). Pain-free period was significantly different between two groups (1.25 h versus 18.73 h) (P<0.001).

***Conclusion: ***The results show that prescription of intratechal buprenorphine prolongs the duration of analgesia without any significant considerable side effects.

The increasing cesarean section rate is a global issue in the developed and developing countries (1). It has shown a rapid and significant rise during the past 30 years. The statistics from the Ministry of Health and Medical Education of Iran reported that about 40.7% of all deliveries are cesarean section all throughout (2). Because of the increasing risks associated with anesthesia in pregnant women, there is a greater tendency toward neuroaxial anesthesia in cesarean section (3). Spinal anesthesia is the method of choice in cesarean section (4). Post cesarean delivery pain relief is important. Good pain relief will improve mobility and can reduce the risk of thromboembolic disease, which increases during pregnancy. It is necessary that pain relief be safe and effective not interfering with the mother’s ability to move around and care for her infant, without leading to any adverse neonatal effects. The most commonly used modalities are systemic administration of opioids, either by intravenous or intramuscular injection, and intratheraceal injection of opioid as part of a regional anesthetic for cesarean delivery (5).

It is proposed that by adding a small dose of opioid to local anesthetic solution, the duration of analgesia can be significantly prolonged without increasing side effects. Intratecal narcotics enhance the sensory blockade of local anesthetics without affecting the sympathetic activity (6). In this method, the annoying complaints like numbness and immobility decrease after recovery. Adding opioid to local anesthetics reduces pain severity during and after surgery and reduces the necessary doses of anesthetic (7). 

Buprenorphine is a long-acting, lipid soluble, mixed agonist-antagonist opioid that has been used in clinical practice since 1979. Buprenorphine is a thebaine derivative with a partial agonist activity at the μ-opioid receptor. Buprenorphine is administered via intravenous, intramuscular, sublingual, and intrathecal routs. It has been used for the treatment of acute/chronic pain and also as a supplement drug in anesthesia. Since buprenorphine dissociates slowly from μ-opioid receptor, it has long duration of action and less addiction potential (8). Like other agonist-antagonist opioids, buprenorphine cannot be used as a single anesthetic. Lidocaine is the most common used local anesthetic. Lidocaine has an extensive use in spinal anesthesia and with widespread popularity. It is a known local anesthetic for spinal anesthesia among obstetric patients (9).

The aim of this study was to evaluate the efficacy and the adverse effects of intrathecal buprenorphine in cesarean section while there is a paucity of published literature assessing buprenorphine when prescribed intrathecally.

## Methods

From June 2010 to December 2012 all patient candidates for elective cesarean section and in ASA class I (American Society of Anesthesiology) aged 17-41 years were included for this study. The patients with local infection at the site of lumbar puncture, any contraindication for lumbar puncture, disorders of the spine, alcoholic patients or those with history of drug abuse, collagen vascular disease, NSAIDs or corticosteroid use, psychotic problems, bleeding disorders, space occupying lesions of the brain, fetal or maternal contraindications for spinal anesthesia, severe fetus distress, blood pressure more than 140/90 mmHg, gestational diabetes, cardiopulmonary diseases and a height of less than 150 cm were excluded. The main variable in this study was pain and the sample size was calculated on the basis of mean differences of pain perception (VAS score) in other studies. Based on clinical experience and review of literature, an educated guess was made that a difference in pain score about 1-2 according to VAS scale between two groups would be statistically significant. Using the data and assuming a study with 90% power and probability of making a type I error of 5%, a sample size of four hundred patients was required to obtain the statistical significance. So, assuming the equal distribution of patients in both groups, four hundred and forty-two healthy full term pregnant women incorporated in the study were randomly divided into two groups using a computer-generated randomization table (simple random sampling) according to their profile number:case group (lidocaine and buprenorphine) and control group (lidocaine). 

The explanation regarding the procedure and study, education regarding VAS score, and necessary written informed consent was done during the preoperative checkup at visit. 

Standard ASA fasting guidelines were followed by all patients upon arrival at the operation theatre, intravenous access was established with an 18G intravenous cannula in a large vein of forearm, and ringer or normal saline (0.9%) was infused before anesthesia (300-500 ml) and vital signs (pulse oximetry, blood pressure and heart rate) were measured and recorded. Then the patients were positioned into a sitting position with the help of a nurse and pulse-oximeter was connected. Dural puncture was done after prep in L3-L4 or L4-L5 levels and after assuring about the CSF, anesthetic drug was infused during 10-15 seconds into the subarachnoid space. Then the patients were positioned back to the supine position and ECG monitoring, non-invasive automatic blood pressure evaluation and pulse-oximetry were performed. Spinal anesthesia was performed for all cases. To ensure blinding, the randomly allocated coded syringes of drugs were prepared by a clinical anesthesia resident but did not perform subarachnoid block or record the outcome intraoperative and postoperative period. The investigator and the attending anesthesiologist performing the study were blinded to the content of the drugs contained in each syringe.

In the case group, 65-70 mg (1.3 to 1.4 ml) of 5% lidocaine plus 0.2 ml of buprenorphine was infused and in the control group, 65-70 mg (1.3-1.4 ml) of 5% lidocaine plus 0.2 ml of 0.9% normal saline was infused. Hemodynamic factors like blood pressure and heart rate were recorded before and at 1, 3, 5, 15, 30, 45, 60 and 75 minutes after spinal anesthesia. Severe decrease in hemodynamic elements was treated appropriately. Surgery was started about 4-5 minutes after induction. 1500-1800 ml isotonic serum was given intravenously to patients during the surgery. Neonatal APGAR (Appearance, Pulse, Grimace, Activity and Respiration) score was recorded at the 1st and the 5^th^ minutes after delivery. 

Oxygen (3 L/min) was administered throughout the procedure via nasal cannula. Intraoperative fluid management was done in relation to body weight of the patient, vital signs, and intraoperative losses. At the end of the surgery, the patients were transferred to the recovery room and monitored by pulseoximetery and NIBP (non invasive blood pressure). Postoperatively, all patients in the study were visited daily and were asked for the presence of a headache and any accompanying symptoms. The pain onset was recorded according to the visual analog scale (VAS) with facial expression (10, 11). Based on this score, no, mild, moderate, severe and worst imaginable pains were measured. 

If there was a moderate to severe pain, 0.5 mg/kg IV pethidine was administrated. In the ward, if there was a moderate pain diclofenac suppository (100 mg) was given, in case of, severe pain or no response to the suppository, Intramuscular pethidine was prescribed. All patients were under observation with regard to probable buprenorphine side effects. This study was registered in the Iranian Registry of Clinical Trials of the Ministry of Health (a branch of World Health Organization) by IRCT2013022112552N1.

Data were collected and analyzed using SPSS Version 16. For the categorical variables, chi-square test or Fisher’s exact test was used (VAS score, APGAR). The mean of continuous data were compared between the two groups using Mann-Whitney and ANOVA tests (age, hemodynamic parameters). We also used t-test for comparing the data between two groups as it was necessary (pain free period, analgesic consumption). The significance level was defined as a p-value less than 0.05.

## Results

**Figure1:**
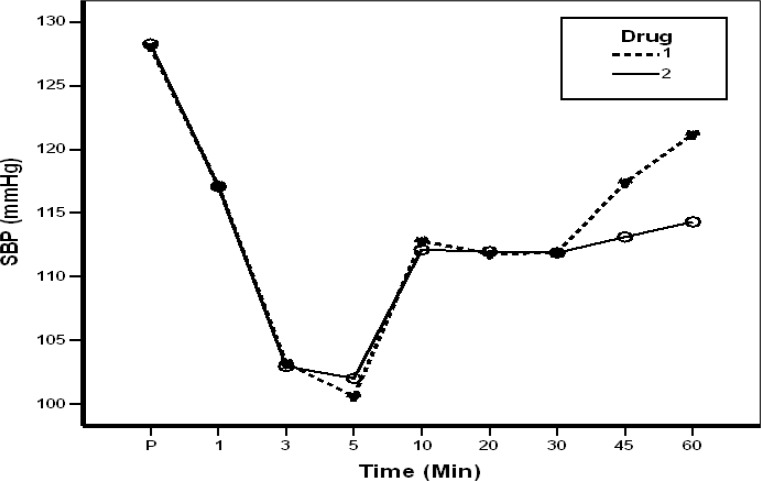
Systolic blood pressure changes in the two studied groups

**Figure 2:**
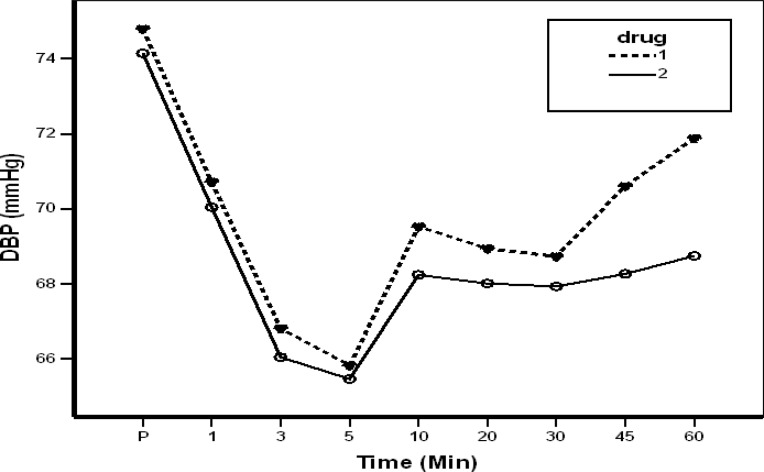
Diastolic blood pressure changes in the two studied groups.

Two hundred eight cases (case group) and 243 subjects (the control group) were selected. The mean age of the case group was 24.4±5.38 and in the control group was 26.84±5.42 years (P=0.389). Systolic blood pressure was not significantly different until the 45th minute but it turned to be significant later on (P<0.001). Diastolic blood pressure showed a significant difference at the 15th minute and after the 60th minute. Heart rate changes were significantly different between cases and controls at 5, 15 and after 60th minutes (P<0.001) (table 1). Hemodynamic changes are shown in Figures 1 and 2. Pain-free period was significantly different between the two groups (P<0.001) (1.25 hours in controls and 18.73 hours in cases). The data showed that all 208 patients in the control group received IV analgesia during 24 hours after surgery. Sixty-one patients received one dose of pethidine and 139 received twice. In contrast, 100 patients in the case group received no analgesia, 115 only one prescription of diclofenac suppository (100 mg) and 18 cases, one dose of IV pethidine.

Nausea vomiting and itching were not significantly different between the two groups. APGAR score was not significantly different between the two groups (P=0.154).

**Table 1:** Comparing mean±SD of heart rate between cases and controls

**Group** **Minutes after spinal anesthesia**	**Mean** **±** **SD**		**P-value**
	**Case**	**control**	
0	109±28	113±23	0.110
1	127±23	124±19	0.145
3	121±19	118±26	0.176
5	131±17	136±19	0.005
15	113±24	105±27	0.001
30	101±16	96±23	0.010
45	98±25	94±19	0.065
60	84±17	97±25	0.000
0	89±20	108±23	0.000

## Discussion

In this clinical trial study, the efficacy and complications of intrathecal buprenorphine in elective cesarean section were evaluated. It has been shown that the mean of pain-free period in the study group (intrathecal lidocaine plus bupernorphine) was 17.65 hours more than the control group (plain intrathecal lidocaine), in which most of these patients did not have any pain during the first 24 hours or the pain was resolved by diclofenac suppository. Other studies showed that adding opioids to local analgesic is an acceptable method to do spinal anesthesia. Prolonged of pain-free period after cesarean section is one of the advantages of spinal anesthesia (7, 12). The present results showed that the supplementation of spinal lidocaine with buprenorphine significantly prolonged the sensory block and postoperative analgesia compared with plain intrathecal lidocaine without any effects on the onset time of sensory block in cesarean section. The safety of intrathecal buprenorphine in caesarean section and its efficacy for postoperative analgesia has been shown in an investigation, too (13). Khan et al. compared analgesia after spinal anesthesia between fentanyle plus bupivacaine and bupivacaine plus buprenorphine and bupivacaine alone. They concluded that adding buprenorphine to bupivacaine could induce longer pain-free periods (6).

Candidio et al. reported that adding buprenorphine to a local analgesic could increase the pain- free period three-times in the method of brachial plexus block (14). In a study in Iran showed that analgesia with lidocaine plus buprenorphine was so much longer than lidocaine alone and no hemodynamic changes were seen in both groups (15). Johnson et al. evaluated the different patients under surgery (laparatomy, gynecology and cardiac surgery) with regard to the adverse effects of buprenorphine. They observed that nausea vomiting and lightheadness were much prevalent with buprenorphine, but the other side effects like decrease in respiratory rate and sleepiness were not different compared to other opioids. 

Higher doses of intrathecal bupernorphine (0.3-0.9 mg) were associated with low side effects and few more advantages like duration of effect or quality of analgesia. Intrathecal bupernorphine could induce 12-24 hours analgesia. As to compare buprenorphine (0.6 mg) with methadone (20 mg) during hysterectomy, the patients who received buprenorphine requested for less analgesia and had longer pain-free period (16).

In the present study, changes in heart rate and blood pressure were significantly different between cases and controls after the 60^th^ minute and more remarkable in controls which showed the back pain in those who received lidocaine alone.

It has been shown that spinal anesthesia for cesarean section is associated with hypotension in mother. Turhanoglu et al. designed a study to evaluate the advantage of intrathecal administration of low dose bupivacaine in cesarean section. They showed that adding bupivacaine (4 mg) to fentanyl (25 mg) did not prevent hypotension but reduced its severity and the dosage needed for treatment by ephedrine (17). It is also reported that the risk of hypotension in mother during cesarean section with local anesthesia can be diminished by the administration of ephedrine or phenylephedrine IV or rise in blood volume with crystalloid or colloids (18).

In the present study, no significant differences were seen between two groups regarding apnea, nausea vomiting and itching. Epidural buprenorphine was used in a study on those with multiple rib fractures and more analgesia and early recovery was reported without significant effects on cardiovascular systems or inducing nausea vomiting and itching (19).

This investigation showed that using intrethecal buprenorphine in cesarean section prolongs the duration of analgesia without any significant changes in hemodynamic status, respiratory problems, side effects like nausea, vomiting and itching.

## Notes

**Funding: **This research project was funded by Babol University of Medical Sciences.
